# Quinine in Epistaxis

**Published:** 1875-04

**Authors:** 


QUININE IN EPISTAXIS.—A writer in the *Lancet* says quinine is *the* remedy in epistaxis. He says that he has tried it more than twenty times, often in aged people, and has never found it to fail.

